# Antitumor Effects of Poplar Propolis on DLBCL SU-DHL-2 Cells

**DOI:** 10.3390/foods12020283

**Published:** 2023-01-07

**Authors:** Xiaoqing Liu, Yuanyuan Tian, Ao Yang, Chuang Zhang, Xiaoqing Miao, Wenchao Yang

**Affiliations:** 1 College of Animal Science (College of Bee Science), Fujian Agriculture and Forestry University, Fuzhou 350002, China; 2 Bee Product Processing and Application Research Center of the Ministry of Education, Fuzhou 350002, China

**Keywords:** propolis, diffuse large B-cell lymphoma, SU-DHL-2 cell line, proteomics, ferroptosis

## Abstract

Propolis is resinous natural product produced by Western honeybees using beeswax and plant and bud exudates, which has a wide range of biological activities, including antioxidation, antibacterial, anti-inflammation, immune regulation, antitumor, and so on. Diffuse large B-cell lymphoma (DLBCL) is an aggressive cancer, and accounts for about 30% of all lymphomas. The effect of poplar propolis on DLBCL has not been reported. The IC_50_ of propolis on the proliferation of DLBCL SU-DHL-2 cell line and its proteins and gene expressions were detected by CCK-8 kit, label-free proteomic, and RT-PCR. The results showed that the IC_50_ of propolis at the 5 × l05/mL cell for 24 h was 5.729 μg/mL. Label-free-based proteomics analysis showed that there were 115 differentially expressed proteins (61 up-regulated and 54 down-regulated proteins) between IC_50_ dose-treated and solvent control groups. There were 32.47% differential proteins located in the nucleus, 20.78% in the cytoplasm, and 14.29% in mitochondria. The most significant different pathway (*p* = 0.0016) of protein enrichment was ferroptosis (including glutamate–cysteine ligase regulatory subunit, ferritin, and heme oxygenase). The relative expression trend of 17 of the total 22 genes selected according to proteomics results was in line with their encoded protein. The highest protein–protein interaction was serine/threonine-protein kinase PLK, which interacted with 16 differential proteins. In conclusion, poplar propolis inhibited SU-DHL-2 cells via ferroptosis pathway, accelerating cell death and down-regulated serine/threonine-protein kinase PLK1, affecting apoptosis of cell. This result provides a theoretical basis for the treatment of DLBCL using propolis.

## 1. Introduction

Propolis, a kind of colloidal solid with an aromatic smell, is processed by Western honey bees after collecting resin from plant or tree and mixing with the secretions of their maxillary glands and wax glands. At present, more than 800 compounds have been isolated and extracted from propolis, and most of them are secondary plant metabolites [1]. Its chemical composition is mainly composed of resin (70%), wax (10%), volatile substances (1%), and other organic compounds, including phenolic compounds, esters, flavonoids, terpenes, beta steroids, aromatic aldehydes, alcohols, vitamins, such as vitamins B1, B2, B6, C, and E, minerals, such as magnesium, calcium, potassium, sodium, copper, zinc, manganese, and iron, and enzymes, such as succinate dehydrogenase, glucose-6-phosphatase, adenosine triphosphatase, and acid phosphatase [1,2,3]. However, different propolis have different chemical compositions depending on its plant origin, regions, and age [3]. Propolis has a wide range of biological activities, such as antibacterial, anti-inflammatory, antioxidant, antitumor, and immune regulation, and has been used in many fields, including food, functional food, medicine and cosmetics, etc. [4].

Among these activities of propolis or its component, antitumor activity, including cancer cell from colorectal, lung, breast, melanoma, gastric, lymphoma, and tongue [5,6,7,8,9,10,11,12,13,14,15,16], is one of the most important biological properties. It was reported that red propolis could reduce pre-tumor lesions, the level of oxidative stress, and the number of abnormal crypt lesions, thereby protecting the colon of rats [5]. The combination of propolis and 5-fluorouracil decreases Cox-2, iNOS contents and reduced the number of abnormal crypt foci and pathological lesions in BALB-c mice [6]. Propolis also has selective cytotoxic effects on human lung cancer cells [7] and tumor cells [8]. Propolis (from India) has an antitumor effect against Dalton’s lymphoma-bearing mice [9]. Some components in propolis have antitumor activity. Pinobanksin and some of its ester derivatives in propolis inhibit the B-cell lymphoma cell (M12.C3.F6) [10]. Galangin, a common flavonoid in propolis, significantly induced melanoma cell apoptosis and inhibited melanoma cells in vitro [11]. Caffeic acid phenethyl ester (CAPE) and artepillin C in propolis played an antitumor function on carcinoma, malignant melanoma, colorectal adenocarcinoma, liver, lymphoma, and neurofibromatosis cancer cells [12,13,14,15,16]. Artepillin C indirectly killed human and murine malignant tumor cells in vitro and in vivo by activating the immune system [12] and inhibited the proliferation of human colon cancer cells via inducing G0/G1 cell cycle prolongation [13]. It also inhibited the growth of mouse nerve fiber xenografts by blocking the PAK1 signaling pathway [14]. CAPE completely inhibited the DNA replication of breast cancer cells at a concentration of 10 μg/mL [15]. CAPE demonstrated antitumor activity on cutaneous T-cell lymphoma by modulating the expression level of key transcription factors [16]. The antitumor effect of polar propolis against diffuse large B cell lymphoma was not yet reported.

Diffuse large B cell lymphoma (DLBCL) represents approximately 30–40% of all cases in different geographic regions, and the most common type of non-Hodgkin lymphoma worldwide. According to the different gene expression profiles, DLBCL can be divided into germinal center B-cell and non-germinal center B-cell types [17]. Approximately 60–70% of patients were cured with the standard therapy, which is rituximab plus cyclophosphamide, doxorubicin, vincristine, and prednisone (R-CHOP) [18]. Kinds of tumor suppressors had inhibition effects on DLBCL. Fbw7, a substrate recognition element of the evolutionarily conserved SCF-type ubiquitin ligase complex, mediates apoptosis through targeting Stat3 for ubiquitylation and degradation in activated B-cell (ABC)-like subtype DLBCL [19]. B-AP15 inhibits cell migration and induces apoptosis in germinal center B-cell-like (GCB) and ABC-DLBCL cells [20]. Realgar, a Chinese traditional medicine, inhibits DLBCL cell growth and induces cell apoptosis mainly by up-regulation of Caspase-3 and BAX expression and down-regulation of BCL-2 expression [21]. Apoptosis in DLBCL SU-DHL-4 cells was induced by 17-dimethylaminoethylamino-17-demethoxygeldanamycin, which can induce oxidative stress and then inhibit the expression of HSPA5 and Bcl-2 but promote the expression of Bax [22]. Overexpression of miR-195 caused the down-regulation of *IL-10, PD-1+T*, and *PD-L1* and increased the secretion of IFN-γ and TNF-α [23]. Although many studies have been carried out on the antitumor activity of propolis on kinds of cancer cells, the study of propolis from China on diffuse large B-cell lymphoma cells has not been reported yet. This study aimed to investigate the effect of propolis on the SU-DHL-2 cells in vitro. Cell proliferation, apoptosis, changes in protein expression, the related gene expression trends, and conduction pathway were determined.

## 2. Materials and Methods

### 2.1. Propolis Extraction and Components Determination

Crude poplar propolis was harvested in Qinglong Manchu Autonomous County, Qinhuangdao, Hebei, China. The extraction of crude propolis and its components determination were performed as in our previous report [24]. Briefly, raw propolis was extracted using ethanol (ratio (w/v) of propolis and 70 ethernol was 1:7.5) under the assistance of ultrasonic wave (of 40 kHz, 20 min, and 60 °C for 3 times) and soaking at room temperature for 2 days. The supernatant was partially evaporated under low pressure after centrifuge (4000× *g* for 10 min). Then, the extract was stored at 4 °C to solidify and remove beeswax on the surface.

### 2.2. Determination of IC_50_ of Propolis Extract on SU-DHL-2 Cell

SU-DHL-2 cell (ATCC CRL-2956, purchased from Cellcook, Guangzhou, China) was cultured with the medium composed of 89% the modified RPMI-1640 basal medium (purchased from Wuhan Prosper Life Technology Co., Ltd. Wuhan, China), 10% fetal bovine serum (purchased from Cellmax Bio Co., Ltd. Lanzhou, China), and 1% penicillin and streptomycin mixture (purchased from HyClone Biochemical Products Co., Ltd. Shanghai, China) in a 5% CO_2_ humidified incubator at 37 °C (C150, Binder, Tuttlingen, German).

Propolis extract was dissolved in DMSO at a concentration of 2 mg/mL. Propolis solution was diluted with a complete medium for 100, 50, 25, 12.5, 6.25, 5, and 2 µg/mL. A complete medium added DMSO (0.25%, *V/V*; equal to DMSO in 100 ug/mL propolis group) was designed as a negative control. They were used to culture SU-DHL-2 cells at a beginning concentration of 5 × 10^5^ cells/mL. Then, the cell suspensions were collected after 24 h to determine cell viability by CCK8 kit (Purchased from DOJINDO, Kumamoto, Japan) at 450 nm using a microplate reader (1510, Thermo Fisher Waltham, MA, USA). IC_50_ of propolis extract on SU-DHL-2 cell for 24 h was calculated using Graphpad Prism 8.4.3 for Windows (GraphPad Software, Inc., La Jolla, CA, USA).

### 2.3. Different Proteins of Different Group Cells by Proteomics

Cells were cultured with IC_50_ propolis (5.729 µg/mL) and solvent control (0.143 µL/mL DMSO in complete medium) at the same condition. After cultivation for 24 h, the cells were collected and snap frozen in liquid nitrogen, and then stored at −80 °C. Their total proteins were extracted according to the references [25,26]. The concentration of total proteins was determined by Coomassie brilliant blue staining [27]. The spectra of proteins in SU-DHL-2 cells were determined using LC-MS-MS with a Q Exactive HF-X mass spectrometer (Thermo Fisher) with a Nanospray Flex™ electrospray ionization (ESI) source by Novgene Biotech Co., Ltd., Beijing, China.

### 2.4. Detection of Relative Gene Expression 

The gene expressions coded key proteins in protein interactions in the ferroptosis pathway and cell cycle (GCL, HO-1, FTH1, HSP70, p62, PPP4R3C, BUB1B, Cyclin B, PLK1) and randomly selected differential proteins (including 9 up-regulated proteins: POLG2, UGDH, NQO1, PPP1CC, TNFRSF10B, ALG8, NADK2, TANK, NDUFS3, and 4 down-regulated proteins: RASSF6, TK1, VNN3, CTU1) were determined using RT-PCR technology. The *β-actin* was used as the internal reference gene, and primers were designed via NCBI’s free online primer design platform. The primer sequences were listed in Supplementary Table S1. The RT-PCR procedure was performed as per our previous report [24] using Real-Time PCR detection by C1000 Touch Thermal Cycler (BIORAD).

### 2.5. ROS Staining

Total intracellular ROS was determined by staining cells with dichlorofluorescin diacetate using the Reactive Oxygen Species Assay Kit (Beyotime Biotechnology) [28]. Staining of cells was observed and taken photograph under an inverted fluorescence microscope (TS-100f, Nikon).

### 2.6. Statistical Analysis

All experiments were performed in triplicate. The experimental results were analyzed using Graphpad Prism 8.4.3 for Windows (GraphPad Software, Inc. San Diego, CA, USA) and expressed as mean ± standard error. Percentages (p) were transformed to arcsin (degree) values (according to the formula: arc sin√p) prior to ANOVA. One-way ANOVA analysis was used to analyze the significance of differences (*p* < 0.01: extremely statistically significant differences between the treatment group and the control group, *p* < 0.05: statistically significant differences). The relative gene expression was represented by the ratio of expression of a gene in propolis-treated cells to that of control cells [29].

The spectra obtained from LC-MS/MS was searched against the uniprot database by Proteome Discoverer 2.2 (Thermo) with a credibility of more than 99% Peptide Spectrum Matches. The identified protein contains at least 1 unique peptide and false discovery rate is less or equal to 1%. The protein quantitation results were statistically analyzed by T-test using Graphpad Prism 8.0.2 for Windows. The proteins whose quantitation significantly different between experimental and control groups (*p* ≤ 0.05 and Fold change (FC) ≥ 2 or FC ≤ 0.5), were defined as differential proteins. All differential proteins were sent to the Gene Ontology database (http://www.geneontology.org/ (accessed on 16 November 2022)) to calculate the number of proteins in each term. Hypergeometric test was applied to find GO entries that were the highest significantly enriched in different proteins compared with all protein backgrounds. Kyoto Encyclopedia of Genes and Genomes (KEGG) was employed to analyze the pathway (http://www.genome.ad.jp/kegg/ (accessed on 16 November 2022)). The protein–protein interactions of differential proteins were performed using the String-db server (http://string.embl.de/ (accessed on 16 November 2022)), in which the minimum required interaction score was medium confidence (0.500). Then, data exported from string-db were loaded into the Cytoscape software (Version 3.9.1; JAVA: 11.0.6 by AdoptOpenJDK) to beautify the PPI network diagram.

## 3. Results

### 3.1. Chemical Composition of Ethanol Extracts of Propolis

There are 51 compounds (Supplementary Table S2) identified from ethanol extracts of propolis (EEP) by UPLC-ESI-MS according to the retention time and secondary mass spectrometry fragmentation of the identified compounds verified through the literature using the same chromatographic and mass spectrometry conditions. Among the 51 identified compounds, there are 31 flavonoids, including luteolin, quercetin, apigenin, breasol kaempferol, etc., 12 phenolic compounds, including p-hydroxybenzoic acid, caffeic acid, p-Hydroxycoumaric acid, isoferulic acid, ferulic acid, etc., 6 kinds of acylated glycerol, and 2 kinds of fatty acid compounds.

### 3.2. The Inhibition Effects of EEP on SU-DHL-2 Cell

**Figure 1:**
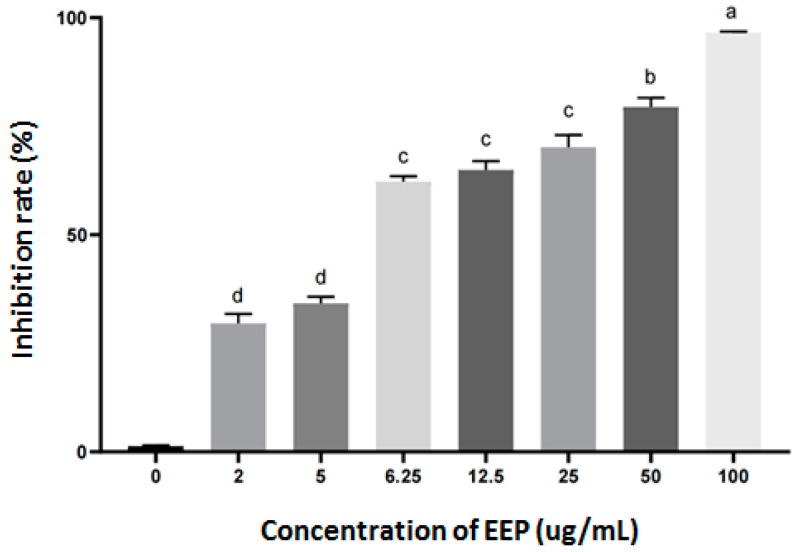
Inhibition rates of SU-DHL-2 cell proliferation by different concentrations of EEP for 24 h. Different letters indicate statistical differences between groups (*p* < 0.05).

Different concentrations of EEP showed different antitumor effects against SU-DHL-2 cells. The inhibition rate of EEP on SU-DHL-2 cells for 24 h treatment was shown in Figure 1. The IC_50_ of EEP on SU-DHL-2 cells for 24 h was 5.729 µg/mL.

### 3.3. Differential Proteins of SU-DHL-2 Cells Treated by EEP and Control

**Figure 2:**
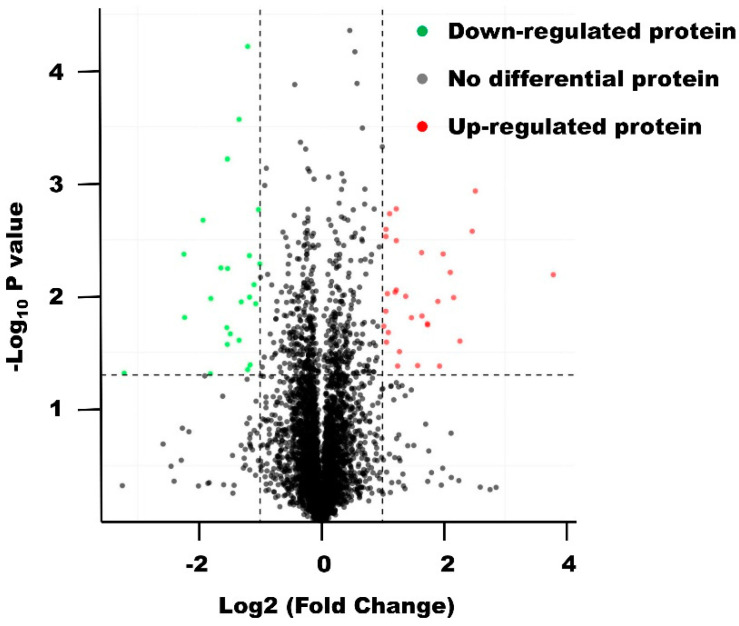
Volcano plot of proteins in SU-DHL-2 cells treated with EEP versus control groups.

Differential proteins were screened according to FC > 2.0 or FC < 0.5 and *p* < 0.05 (EEP vs. control groups). There were 61 up-regulated proteins, and 54 down-regulated proteins (partly differential proteins (*p* < 0.01) were shown in Supplementary Table S3). Volcano plot of proteins in SU-DHL-2 cells of the two groups was shown in Figure 2.

The subcellular localization results of the differential proteins showed 32.47% nucleus protein, followed by 20.78% cytoplasm protein, 14.29% mitochondrion protein, 6.49% lysosome proteins, 6.49% endoplasmic reticulum protein, 3.9% plasma membrane protein, 3.90% Golgi apparatus protein, 3.90% extracell protein, 3.90% cytoskeleton protein, 1.30% microsome protein, 1.30% endosome protein, and 1.30% centrosome proteins.

These differential proteins played functions in different pathways. The significant differential proteins in these pathways (*p* < 0.05), which were separately analyzed in up-regulated and down-regulated proteins, were shown in Table 1.

**Figure 3:**
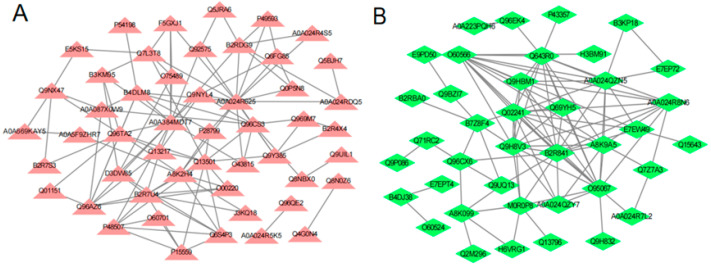
Interaction of differential proteins: (**A**) interaction diagram of differentially up-regulated proteins; (**B**) interaction diagram of differentially down-regulated proteins. The triangles or diamonds represent proteins, straight lines represent the interaction relationship between proteins.

All the interactions of differential proteins were shown in Figure 3. Among the interactions of up-regulated proteins, heat shock protein 70 (HSP70) was the most interacted protein with 13 proteins. Among the interactions of down-regulated proteins, serine/threonine-protein kinase PLK (PLK1 or PLK) was the most interacted protein with 16 differential proteins.

### 3.4. RT-PCR Identification

**Figure 4:**
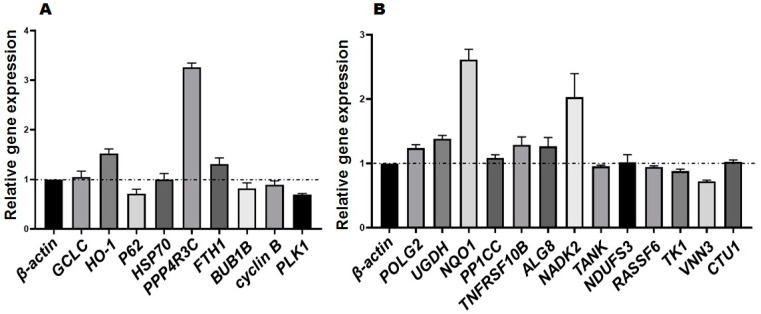
Relative gene expression: (**A**) genes coded differential proteins screened from ferroptosis pathway, cell cycle pathway and protein interaction; (**B**) genes coded differential proteins were randomly selected. Genes with expression greater than 1 were up-regulated and less than 1 were down-regulated.

The results of relative gene expression were shown in Figure 4. The expressions of *GCLC*, *HO-1*, *PPP4R3C*, and *FTH1* were up-regulated; the relative expressions of *BUB1B*, *Cyclin B*, and *PLK1* were down-regulated. They are consistent with the protein expression trend with exception of *p62* and *HSP70* genes. The relative expression of *POLG2, UGDH, NQO1, PPP1CC, TNFRSF10B, ALG8*, and *NADK2* were up-regulated, and that of *RASSF6, TK1*, and *VNN3* were down-regulated. These gene expressions trends were consistent with the coded protein expressions trends with exception of *HSP70, NDUFS3,* and *CTU3* genes.

### 3.5. ROS Staining of SU-DHL-2 Cell

**Figure 5:**
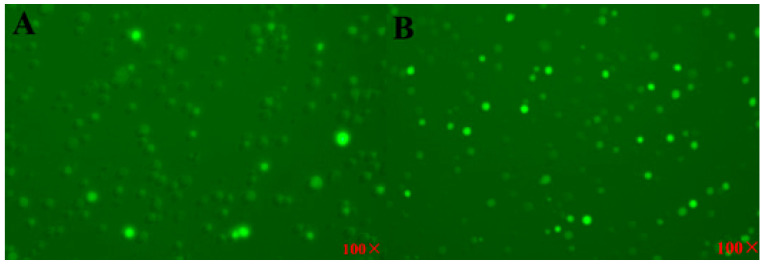
ROS staining of SU-DHL-2 cells: (**A**) control group; (**B**) treatment group, propolis at IC_50_ (×100).

The results showed that the cells treated with EEP have more ROS activity than that of control cell. The photographs of ROS-stained cell are shown in Figure 5.

## 4. Discussion

Among the 51 identified compounds of EEP, there are 31 flavonoids, including luteolin, quercetin, apigenin, breasol kaempferol, etc., 12 phenolic compounds, including p-hydroxybenzoic acid, caffeic acid, p-Hydroxycoumaric acid, isoferulic acid, ferulic acid, etc., 6 kinds of acylated glycerol, and 2 kinds of fatty acid compounds. The components of EEP used in this experiment were more than others, which were 20 components [30], 31 components [31], 49 components [24] in EEP (poplar) harvested in Yunnan, Shandong, and Shandong, China, respectively. These differences in chemicals and quantities were caused by the region, plant source, and age of propolis [32].

Different propolis have different antitumor activities. It was reported that EEP from three different sources had selective cytotoxicity, and the IC_50_ on tongue cancer cells treated for 24 h were about 88 µg/mL, 110 µg/mL, and 150 µg/mL, respectively [33]. The IC_50_ of propolis extract (nano-vesicular formulation of propolis) on A549 lung cancer and BEAS-2B healthy lung cells were 25.44 ± 4.97 μg/mL and 55.68 ± 6.24 μg/mL [34]. The IC_50_ of EEP samples from different regions in Thailand against A549 cells were 106 ± 0.004 µg/mL, 199 ± 0.009 µg/mL, and 87 ± 0.012 µg/mL, and for Hela cells were 81 ± 0.006 µg/mL, 116 ± 0.023 µg, and 54 ± 0.005 µg/mL, respectively [35]. The IC_50_ of hydroalcoholic Brazilian red propolis on Hep-2 cells was 145.40 ± 6.56 µg/mL after treatment for 24 h on Hep-2 cells [36]. Chinese propolis water extract and its effective components induced apoptosis of breast cancer cells (MCF-7, MDA-MB-231, A549, and HeLa cells) by inhibiting tumor cell migration, activating caspase 3, and promoting ROS production when the concentrations of propolis and each of its effective components (pinobanksin, caffeic acid benzyl ester, caffeic acid phenethyl ester, apigenin, pinocembrin, chrysin, and galangin) were 100 µg/mL and 80 μM [37]. Massive apoptosis was found in human lymphocytic leukemia cells treated with 10 µM CAPE [38]. The IC_50_ of EEP against SU-DHL-2 cells was 5.729 μg/mL (Figure 1), which is far lower than these reports. Propolis exhibits different cytotoxicity to different tumor cell lines, which may be related to the cell concentration and sensitivity to propolis, extraction method, compounds, and duration of action of propolis.

The antitumor mechanism of propolis waved because of different types of cancers or their cell lines, different plant sources of propolis, and the delivery system of the drug. The proteomic technology was widely used to determine the differential proteins in different treatment cells. There were 115 differential proteins between EEP-treated and control SU-DHL-2 cells, which engaged in different pathways to inhibit the proliferation of the cancer cell (Table 1). The highest significant pathway was ferroptosis (*p* = 0.0017), which was also found in antitumor effects on other cancer cells [39]. This result was identified by the result of ROS staining (Figure 5). The ferroptosis pathway includes differential proteins of glutamate-cysteine ligase regulatory subunit (GCLM), ferritin (FTH1), and heme oxygenase (HO). GCLM might control the glutathione (GSH) levels and increase the efficiency of γ- glutamyl-cysteine synthesis in response to oxidative stress [40]. Then, higher GSH content in cancer cells with the antitumor drugs had higher cell growth inhibition potencies [41]. It was reported that ferritin enhanced apoptosis of non-small cell lung cancer cells through the modulation of the miR-125b/p53 axis [42]. It also had antigrowth effects in breast cancer cells by inhibiting the expression of c-MYC [43] and ovarian cancer stem cells [44]. HO has HO-1, HO-2, and HO-3 isoforms, of which HO-1 and HO-2 catalyze the heme into biliverdin, iron, and carbon monoxide. Overexpression of HO-1 is in response to substrate heme, proinflammatory cytokines, reactive oxygen species (ROS), nitric oxide (NO), metalloporphyrins, heavy metals, prostaglandins, UV irradiation, and others [45]. HO-1 induced ferroptotic cell death of HT-1080 fibrosacoma cells [46], human breast cancer, and lung cancer cells by mediating BAY 11–7085 [47], human colon cancer cells [48], and eradicated high-risk neuroblastoma [49]. Our results (Table 1) showed that HO-1 in the ferroptosis pathway induced the death of SU-DHL-2 cells treated by EEP. Proteins of ferritin and heme oxygenase were also involved in mineral absorption pathway (*p* = 0.0076), which is a common pathway in antitumor reports, such as colorectal cancer [50], colon cancer cells [51], prostate cancer [52], etc. Heme oxygenase also plays an important antitumor effect in fluid shear stress and atherosclerosis pathway (*p* = 0.0108). Ferritin was also involved in necroptosis pathway (*p* = 0.0321).

Another significant pathway is the fluid shear stress and atherosclerosis and ubiquinone pathway, which includes sequestosome-1, which is also involved in necroptosis pathway (*p* = 0.0321), NAD(P)H dehydrogenase [quinone] 1, and heme oxygenase. Sequestosome-1, which is also called p62, is a multidomain, multifunctional protein, which is involved in autophagy, defense against oxidative stress via activation of the Keap1/Nrf2 system, protein aggregation and sequestration, and apoptosis for different types of cancer cells [53,54,55]. NAD(P)H dehydrogenase [quinone] 1 was also involved in Ubiquinone and other terpenoid-quinone biosynthesis pathways (*p* = 0.0247). This NAD(P)H quinone dehydrogenase 1 could maintain p53 stability for breast cancer cells [56]. Tumor necrosis factor receptor superfamily member 10A is one of the tumor necrosis factors involved in the necroptosis pathway. It is reported that tumor necrosis factor played an important role in apoptosis in an ovarian cancer cell line [57]. Pathways of influenza A, ubiquitin-mediated proteolysis, ascorbate and aldarate metabolism, and antigen processing and presentation also played an important role in the cancer cell death process.

Apart from the up-regulation pathway, down-regulation pathways of cell cycle, progesterone-mediated oocyte maturation, foxO signaling, hippo signaling pathway—multiple species—also contributed to the antitumor effect of EEP on SU-DHL-2 cells. Cell cycle kinases were designed as targets of therapeutic drug development for cancer [58,59]. Forkhead box O (FoXO), the subfamily of the fork head transcription factor family with important roles in cell fate decisions, plays a pivotal functional role as a tumor suppressor in a wide range of cancers. Ras association domain-containing protein 6 in the hippo signaling pathway is one of the members of the Ras-association domain family that form the core of a highly conserved tumor suppressor network [60].

Among protein–protein interactions, PLK1 was the most interacted protein. It was reported that the down-regulation of PLK1 induced apoptosis [61], premature senescence [62], and elevation drug sensitivity [63] of breast cancer cells and decreased the viability of cancer cells in endothelial cells [64], cell cycle arrest, and apoptosis of cutaneous T-cell lymphomas [65]. Expression of PLK1-inhibited or siRNA-treated showed antitumor activity against prostate, breast, cervical, colon, and lung cancer cells [66,67,68], etc. PLK1 has a potential application strategy in cancer therapy [66,67,68,69]. Knockdown of *KIF23* or inhibition of KIF23, a secondary high interacted protein with 14 proteins, suppressed the growth of lung cancer cells [70,71], triple negative breast cancer cells (MDA-MB-231 and BT549) [72], glioma [73], etc. It was also reported that patients with a high expression of KIF23 had a poor survival [74]. Hsp70 is a dual-function protein, intracellular Hsp70 suppressed apoptosis and lysosomal cell death, and extracellular Hsp70 promoted tumorigenesis and angiogenesis. Additionally, other evidence showed that intracellular Hsp70 promoted apoptosis, and membrane-associated/extracellular Hsp70 elicited antitumor innate and adaptive immune responses [75]. Other differential proteins also played important roles in the antitumor activity of EEP against SU-DHL-2 cells.

The relative expression trend of genes selected to perform RT-PCR was not absolutely the same as their proteins. Although most autosomal gene replication is carried out at the protein level, the copy-number of 23–33% of proteins in complex proteins were influenced by post-transcriptional regulation [76]. The correlation between protein and mRNA is about 0.5 [77], which is weak for the structurally stable proteins and mRNA expression and relatively poor for less stable mRNA and protein expression in high-grade serous ovarian cancer [78]. It was also reported that about 30% of mRNA transcription is not related to protein expression in breast cancer [79].

There are some defects in this antitumor research using cell line, but the xenograft in nude mice did not succeed many times. This may be caused by fewer cancer stem cells in the SU-DHL-2 cell samples injected in nude mice. The metabonomics, cancer stem cell or xenograft model, deletion genes of key proteins, or other methods can be employed to explore more accurate regulation of EPP antitumor against the SU-DHL-2 cell approach for new drug development.

## 5. Conclusions

The IC_50_ of propolis at the 5 × l0^5^/mL cell for 24 h was 5.729 μg/mL. Its antitumor mechanism was explored by label-free-based proteomics, which showed that there were 115 differentially expressed proteins between IC_50_ treated and solvent control groups, which were 61 up-regulated (53.04%) and 54 down-regulated (46.96%) proteins. The main pathway of protein enrichment was the ferroptosis pathway. The most interacted protein was serine/threonine-protein kinase PLK, which interacted with 16 differential proteins. In conclusion, poplar propolis inhibited SU-DHL-2 cells via ferroptosis pathway, accelerating cell death, and down-regulated serine/threonine-protein kinase PLK, affecting apoptosis of the cell.

## Figures and Tables

**Table 1:** These differential proteins in different pathways.

Pathway	*p*-Value	Description of Proteins
Ferroptosis	0.0016	Glutamate–cysteine ligase regulatory subunit, Ferritin, Heme oxygenase
Mineral absorption	0.0076	Ferritin, Heme oxygenase
Fluid shear stress and atherosclerosis	0.0108	Sequestosome-1, NAD(P)H dehydrogenase [quinone] 1, Heme oxygenase
Ubiquinone and other terpenoid–quinone biosynthesis	0.0247	NAD(P)H dehydrogenase [quinone] 1
Necroptosis	0.0321	Sequestosome-1, Ferritin, Tumor necrosis factor receptor superfamily member 10A
Influenza A	0.0414	DnaJ homolog subfamily C member 3, Epididymis secretory sperm binding protein, Tumor necrosis factor receptor superfamily member 10A
Ubiquitin-mediated proteolysis	0.0431	NEDD8-conjugating enzyme UBE2F, Ubiquitin-conjugating enzyme E2 J1, cDNA, FLJ92255, highly similar to Homo sapiens ring finger protein 7 (RNF7), mRNA
Ascorbate and aldarate metabolism	0.0488	UDP-glucose 6-dehydrogenase
Antigen processing and presentation	0.0491	Epididymis secretory sperm binding protein, cDNA FLJ78235
Cell cycle	0.0201	Mitotic checkpoint serine/threonine-protein kinase BUB1 beta, Serine/threonine-protein kinase PLK, G2/mitotic-specific cyclin-B2
Progesterone-mediated oocyte maturation	0.0387	Serine/threonine-protein kinase PLK,G2/mitotic-specific cyclin-B2
FoxO signaling pathway	0.0430	Serine/threonine-protein kinase PLK,G2/mitotic-specific cyclin-B2
Hippo signaling pathway—multiple species	0.0457	Ras association domain-containing protein 6

## Data Availability

Data are contained within this article.

## Supplementary Materials

The following supporting information can be downloaded at: https://www.mdpi.com/article/10.3390/foods12020283/s1, Table S1: Primers sequence for RT-PCR. Table S2: Components of EEP. Table S3: Partly differential proteins (*p* < 0.01). Refs. [80,81,82,83] cited in Supplementary Materials.
