# Supplementary material for: Antitumor Effects of Poplar Propolis on DLBCL SU-DHL-2 Cells
Source: Foods. 2023 Jan 7;12(2):283. doi: 10.3390/foods12020283 (PMC9857396; doi:10.3390/foods12020283)
Supplement: Supplementary file 1 [file foods-12-00283-s001.zip › foods-2075618-supplementary.pdf]

Supplement table S1

Table S1. Primers sequence for RT-PCR

Primer	Sequnence (5'→3')
β-actin-F	GATCATTGCTCCTCCTGAGC
β-actin-R	ACTCCTGCTTGCTGATCCAC
POLG2-F	TTCTGGTTACGTCATCGACTCC
POLG2-R	GTTTCCTTTCCGGCCTTCTTC
UGDH-F	TTATTTCTGTGCTGTCCAACCCT
UGDH-R	CTGGCCCTCTGGAGTTTCAT
HO-1-F	TCTTGGCTGGCTTCCTTACC
HO-1-R	GGATGTGCTTTTCGTTGGGG
NQO1-F	TGAAAGGCTGGTTTGAGCGA
NQO1-R	TCCAGGCGTTTCTTCCATCC
PPP1CC-F	GACTGGCTTGACCTTTCCCT
PPP1CC-R	TCAACTTCAATTCAGGCCGC
TNFRSF10B-F	TGGTCAAGGTCGGTGATTGT
TNFRSF10B-R	GATGCCTGAGAGAGAACAGGG
ALG8-F	CCCCACATACCATTCCACAGA
ALG8-R	CGTCCACTCTGAAGTTGCCT
NADK2-F	CACTTAACCTCTGCCCGTCC
NADK2-R	TCGGTAGCAAGTCATCGTGG
GCL-F	AGGTCAAACCCAACCCAGT
GCL-R	TGTTAAGGTACTGAAGCGAGG
BUB1B-F	GGATGGGTCCTTCTGGAAACT
BUB1B-R	GTGGCCTCATCATTGGCATTC
RASSF6-F	GGCACTGGTTTCCCTCTACC
RASSF6-R	TTCACCATCGTTGTTCGGCT
TK1-F	GGCACAGAGAAGGAGCAGATT
TK1-R	CAGCCACACAAAGGAGAGTTC
VNN3-F	GTGGTGGTGGTGGATGAGTT
VNN3-R	AATGGAAGGGAACAGCCGAG
FTH1-F	CAGAACTACCACCAGGACTCA
FTH1-R	TCAAAGCCACATCATCGCGG
HSP70-F	GTGTAACCCCATCATCAGCG
HSP70-R	GCTCCAAAACAAAAACAGCAATCT
PPP4R3C-F	TATGACGAGCAGTTCCAGGG
PPP4R3C-R	AGGTATCTGTGACCGCAGGA
P62-F	TACCAGGACAGCGAGAGGAAG
P62-R	ATCCTTTCTCAAGCCCCATGT
TANK-F	AGCATTGTTAGAGCCTGTGGAA
TANK-R	TGAGTTGCTCGCCAATGTTT
NDUFS3-F	GCTTTCCCAGTCTATCGCCA
NDUFS3-R	GCGCTGTCTAGGATCCACAT
CTU1-F	GGAGCGTGACTTGATGGACA
CTU1-R	CCGTGGTGGAGACGAAATGA

Cyclin B-F	GATACTGCCTCTCCAAGCC
Cyclin B-R	GCACACAATTATTCTCAAGTTGTC
PLK1-F	CCTGCACCGAAACCGAGTTA
PLK1-R	ACCTCGAAACTGTGCCCTTT

Supplement table S2

Table S2. Components in EEP

No	tR （min）	λmax (nm)	Selected ion	Elemental composition	Measured mass	Calculated mass	Mass error	MS/MS fragmentation	Identification
1	2.52	236, 279, 313	[M−H]	$C_7H_6O_3$	137.0237	137.0239	0.6	137.0245	p-Hydroxybenzoic acid)[b]
2	3.29	241, 323	[M−H]	$C_9H_8O_4$	179.0341	179.0344	-0.3	179.0341, 135.0443	Caffeic acid[a, b, c]
3	3.94	237, 310	[M−H]	$C_9H_8O_3$	163.0392	163.0395	-0.3	163.0392, 119.0494	p-Coumaric acid[b, c]
4	4.33	243, 323	[M−H]	$C_{10}H_{10}O_4$	193.0498	193.0501	-0.3	193.0498, 133.0291	Isoferulic acid [b, c]
5	4.54	244, 323	[M−H]	$C_{10}H_{10}O_4$	193.0494	193.0501	-0.7	193.0494, 134.0366	Ferulic acid [a, b, c]
6	5.79	265	[M-H]	$C_{21}H_{20}O_{10}$	431.0975	431.0978	-0.3	431.0975, 268.0372, 239.0372, 211.0394, 167.0493	Apigenin-7-O-β-D-glucopyranoside[b]
7	6.81	249, 347	[M−H]	$C_{15}H_{10}O_6$	285.0400	285.0399	0.1	285.0400, 571.0873	Luteolin [a, b]
8	6.90	250, 368	[M−H]	$C_{15}H_{10}O_7$	301.0352	301.0348	0.4	301.0352, 285.0759	quercetin [a, b]
9	7.25	231, 287	[M−H]	$C_{16}H_{14}O_5$	285.0783	285.0763	2.0	285.0783, 267.0668, 252.0437, 239.0722, 138.0326	Pinobanksin-methyl ether [b]
10	7.55	252, 354	[M−H]	$C_{16}H_{11}O_7$	315.0506	315.0505	0.1	315.0506, 300.0273, 271.0244	Quercetin-3-methyl ether [b, c]
11	8.64	266, 340	[M−H]	$C_{15}H_{10}O_5$	269.0453	269.0450	0.3	269.0463	Apigenin [a,]
12	8.92	291	[M−H]	$C_{15}H_{12}O_5$	271.0615	271.0606	0.9	271.0615	Pinobanksin [b, c]
13	9.08	249, 265, 367	[M−H]	$C_{15}H_{10}O_6$	285.0401	285.0399	0.2	285.0401	Kaempferol [a, b]
14	9.29	285	[M−H]	$C_{16}H_{14}O_4$	269.0818	269.0814	0.4	269.0818, 254.0577	Pinobanksin-5-methyl ether [b, c]
15	10.08	266, 292, 347	[M−H]	$C_{16}H_{11}O_6$	299.0556	299.0556	0	299.0556, 284.0321, 271.0608	Kaempferol-3-methyl ether [b, c]
16	10.67	252, 353	[M−H]	$C_{17}H_{14}O_7$	329.0664	329.0661	0.3	314.0426, 299.0193	Quercetin-dimethyl ether [b, c]
17	11.43	310	[M−H]	$C_{16}H_{12}O_5$	283.0603	283.0606	-0.3	283.0363, 239.0363, 211.0405, 167.0523	4-[4-[(1E)-2-Carboxyethenyl]phenoxy]benzoic acid [b]
18	11.70	289	[M−H]	$C_{18}H_{16}O_6$	327.0863	327.0869	-0.6	327.0863, 285.0754, 267.0652	Pinobanksin-5-methyl ether-3-O-acetate [b, c]
19	12.39	253, 366	[M−H]	$C_{16}H_{11}O_6$	315.0507	315.0505	0.2	315.0507, 299.055	Isorhamnetin [b, c]

20	13.85	254, 355	[M−H]	$C_{17}H_{14}O_7$	329.0659	329.0661	-0.2	329.0659, 314.0426, 299.0191, 271.0242	Quercetin-dimethyl ether isomer [b, c]
21	14.89	245, 326	[M−H]	$C_{14}H_{16}O_4$	247.0968	247.0970	-0.2	247.0968, 179.0342, 161.0236, 135.0443	Caffeic acid isoprenyl ester [b, c]
22	15.36	267, 313	[M−H]	$C_{15}H_{10}O_4$	253.0502	253.0501	0.1	253.0502, 143.0496	Chrysin [a, b]
23	15.56	245, 325	[M−H]	$C_{14}H_{16}O_4$	247.0977	247.0970	0.7	247.0977, 133.0289	Caffeic acid isoprenyl ester (isomer [b, c]
24	15.70	245, 326	[M−H]	$C_{16}H_{14}O_4$	269.0815	269.0814	0.1	269.0815, 178.0262, 161.0235, 133.0287	Caffeic acid benzyl ester [b, c]
25	16.31	231, 289	[M−H]	$C_{15}H_{12}O_4$	255.0659	255.0657	0.2	255.0659	Pinocembrin [b, c]
26	16.81	265, 359	[M−H]	$C_{15}H_{10}O_5$	269.0451	269.0450	0.1	269.0451, 143.0493	Galangin [a, b]
27	17.45	289	[M−H]	$C_{16}H_{14}O_5$	285.0765	285.0763	0.2	285.0765, 269.0452	isomer [b, e]
28	17.79	231, 293	[M−H]	$C_{17}H_{14}O_6$	313.0718	313.0712	0.5	313.0764, 283.1015, 271.0629, 253.0555	Pinobanksinr-3-O-acetate [b, c]
29	18.57	265	[M−H]	$C_{16}H_{12}O_5$	283.0608	283.0606	0.2	283.0608, 268.0405	Acacetin [b, c]
30	20.78	310	[M−H]	$C_{16}H_{14}O_3$	253.0866	253.0863	0.3	253.0866, 145.0341, 117.0368	p-Coumaric acid benzyl ester [b, d]
31	21.52	248, 326	[M−H]	$C_{18}H_{16}O_4$	296.0969	295.0970	-0.1	295.0969, 133.0288	Caffeic acid cinnamyl ester[b, c]
32	21.82	245, 325	[M−H]	$C_{18}H_{16}O_4$	297.1118	297.1127	-0.9	297.1118, 179.0340, 161.0235, 133.0286	Caffeic acid phenylpropyl ester [e]
33	22.97	292	[M−H]	$C_{18}H_{16}O_6$	327.0874	327.0869	0.5	327.0874, 253.0507	Pinobanksin-3-O-propionate[b, c]
34	26.87	250, 309	[M−H]	$C_{18}H_{16}O_3$	279.1027	279.1023	0.4	279.1023, 117.0368	*p*-Coumaric cinnamyl ester[b, d]
35	27.82	251, 291	[M−H]	$C_{19}H_{18}O_6$	341.1026	341.1025	0.41	341.1026, 253.0505, 197.0633, 143.0532	Pinobanksin-3-O-butyrate [b, c]
36	28.12	291	[M−H]	$C_{20}H_{18}O_6$	353.1026	353.1025	0.1	353.1026, 253.0523	Pinobanksin-3-O--pentenoate [b, c]
37	28.58	251, 289	[M−H]	$C_{27}H_{24}O_8$	475.1389	475.1393	-0.4	475.1389, 413.1958, 265.1476	Pinobanksin-3-O-acetate-5-O-phydroxyphenylpropionate[b, c]
38	30.28	291	[M−H]	$C_{20}H_{20}O_6$	355.1179	355.1182	-0.3	355.1179, 265.1517, 253.0523	Pinobanksin-3-O-pentanoate or 2-methylbutyrateb [b, c]
39	30.43	291	[M−H]	$C_{20}H_{20}O_6$	355.1179	355.1182	-0.3	355.1179, 253.0523	Pinobanksin-3-O-pentanoate or 2-methylbutyrateb[b, c]
40	30.91	291	[M−H]	$C_{21}H_{20}O_6$	367.1185	367.1182	0.3	367.1185, 315.1601, 265.1478, 253.0523	Pinobanksin-O-hexenoate [b, c]
41	31.32	292	[M−H]	$C_{24}H_{20}O_6$	403.1184	403.1182	0.2	403.1184, 297.2431, 253.0555	Pinobanksin-3-O-phenylpropionate [b, c]
42	32.18	279	[M−H]	$C_{18}H_{30}O_3$	293.2122	293.2117	0.5	293.2122	(10E, 12Z)-9-oxo-, 10, 12-Octadecadienoic acid [f]
43	32.30	291	[M−H]	$C_{21}H_{22}O_6$	369.1338	369.1338	0	369.1338, 309.1790, 253.0523	Pinobanksin-3-O-hexanoate [b, c]
44	32.62	277	[M−H]	$C_{18}H_{30}O_3$	293.2117	293.2117	0.0	293.2117	(10E, 12Z)-9-oxo-, 10, 12-Octadecadienoic acid isomer [f]
45	33.14	290	[M−H]	$C_{26}H_{22}O_6$	429.1335	429.1338	-0.3	429.1335, 271.0696, 253.0588	Brevifolin-3-O-phenylpentenoate [b]
46	34.01	-	[M−H]	C20H40O4	343.2844	343.2848	-0.4		(3R, 8R)-3, 8-Dihydroxyeicosanoic acid [f]
47	35.267		[M+HCOO]	C25H48O8	505.3378	505.3377	0.1	505.3378, 495.3087, 459.3316	3-(Acetyloxy)-2-hydroxypropyl (3R, 8R)-3, 8-dihydroxyeicosanoate [f]

48	36.37	[M+HCOO]	$C_{25}H_{50}O_6$	491.3585	491.3584	0.1	491.3585, 481.3297	2, 3-Dihydroxypropyl (3R, 8R)-3, 8-dihydroxydocosanoate [f]
49	37.44	[M+HCOO]	$C_{26}H_{50}O_7$	519.3533	519.3533	0.0	519.3533, 509.3248	3-(Acetyloxy)-2-hydroxypropyl (3R, 8R)-3, 8-dihydroxyheneicosanoate [f]
50	38.49	[M−H]	$C_{22}H_{44}O_4$	371.3162	371.3161	0.1	371.3162, 326.183	(3R, 8R)-3, 8-Dihydroxydocosanoic acid [f]
51	39.61	[M+HCOO]	$C_{28}H_{53}O_9$	533.3693	533.3690	0.3	533.3693, 523.3404	3-(Acetyloxy)-2-hydroxypropyl (3R, 8R)-3, 8-dihydroxydocosanoate [f]

aConfirmed with standard;

bconfirmed with MSn fragmentation;

cconfirmed with references: Falcão, S.I.; Vale, N.; Gomes, P.; Domingues, M.R.; Freire, C.; Cardoso, S.M.; Vilas-Boas, M. Phenolic profiling of Portuguese propolis by LC–MS spectrometry: Uncommon propolis rich in flavonoid glycosides. *Phytochemical Analysis*. 2013, *24*(4), 309-318.;

[d]Nagaoka, T; Banskota, A.H.; Tezuka, Y.; Midorikawa, K.; Matsushige, K.; Kadota, S.. Caffeic acid phenethyl ester (CAPE) analogues: Potent nitric oxide inhibitors from the Netherlands propolis. *Biological & Pharmaceutical Bulletin*. 2003, 26, 487-491.

[e]Bilikova, K.; Popova, M.; Trusheva, B.; Bankova, V.A. New anti-Paenibacillus larvae substances purified from propolis. *Apidologie*. 2013, 44, 278-285.

[f] Bloor, S.; Catchpole, O.; Mitchell, K.; Webby, R.; Davis, P. Antiproliferative acylated glycerols from New Zealand propolis. *Journal of Natural Products*. 2019, 82, 2359-2367.

Supplement table S3

Table S3. Partly differential proteins ($p < 0.01$)

Gene ID	Description of protein	Regulation
O60566	Mitotic checkpoint serine/threonine-protein kinase BUB1 beta	up
Q13217	DnaJ homolog subfamily C member 3	up
H6VRG1	Keratin 1	up
P58546	Myotrophin	up
Q643R0	HCTP4	up
Q13501	Sequestosome-1	up
B2R841	Serine/threonine-protein kinase PLK	up
Q9HCD5	Nuclear receptor coactivator 5	up
O75489	NADH dehydrogenase [ubiquinone] iron-sulfur protein 3, mitochondrial	up
P28799	Progranulin	up
P48507	Glutamate--cysteine ligase regulatory subunit	up
P15559	NAD(P)H dehydrogenase [quinone] 1	up
O60701	UDP-glucose 6-dehydrogenase	up
O43815	Striatin	up
Q99590	Protein SCAF11	up
A0A024R8N6	Thymidine kinase	up
Q96CS3	FAS-associated factor 2	up
Q96CX6	Leucine-rich repeat-containing protein 58	up
Q71RC2	La-related protein 4	up
Q96TA2	ATP-dependent zinc metalloprotease YME1L1	up
J3KQ18	D-dopachrome decarboxylase	up
Q0P5N8	TMSB4X protein (Fragment)	up
Q9NYL4	Peptidyl-prolyl cis-trans isomerase FKBP11	up
O60524	Nuclear export mediator factor NEMF	up
A0A1B0GUA3	KIF-binding protein	up

Q6S4P3	Ferritin	up
E7EW49	CLIP-associating protein 2	up
A0A024QZY7	Geminin, DNA replication inhibitor, isoform CRA_a	up
Q5JRA6	Transport and Golgi organization protein 1 homolog	up
B2R7U4	Heme oxygenase	up
A8K099	cDNA FLJ77959, highly similar to Homo sapiens protein kinase, cAMP-dependent, regulatory, type I, beta (PRKAR1B), mRNA	up
P30047	GTP cyclohydrolase 1 feedback regulatory protein	up
F8W0Q9	Periphilin-1	up
B4DN80	Peptidyl-prolyl cis-trans isomerase	up
B4DJ38	cDNA FLJ56092, highly similar to Pentatricopeptide repeat protein 1	up
A0A024R7L2	HSPC142 protein, isoform CRA_a	up
Q8NBX0	Saccharopine dehydrogenase-like oxidoreductase	up
P49593	Protein phosphatase 1F	up
A0A024RAY1	Activating transcription factor 7 interacting protein, isoform CRA_a	up
Q13796	Protein Shroom2	up
B2RDG9	cDNA, FLJ96603, highly similar to Homo sapiens actin-related protein 10 homolog (S. cerevisiae) (ACTR10), mRNA	up
Q96M27	Protein PRRC1	up
A0A087X0W9	Deubiquitinase OTUD6B	up
M0R0P8	Unconventional myosin-IXb	up
Q4G0N4	NAD kinase 2, mitochondrial	up
A0A223PQH6	HCFC1	down
Q02241	Kinesin-like protein KIF23	down
E7EPT4	NADH dehydrogenase [ubiquinone] flavoprotein 2, mitochondrial	down
B7Z8F4	cDNA FLJ56523, highly similar to ATP-dependent RNA helicase DHX8	down
P43357	Melanoma-associated antigen 3	down
Q13571	Lysosomal-associated transmembrane protein 5	down
O95067	G2/mitotic-specific cyclin-B2	down
A0A024QZN5	ZW10 interactor, isoform CRA_b	down
Q69YH5	Cell division cycle-associated protein 2	down
Q86WQ0	Nuclear receptor 2C2-associated protein	down
Q99504	Eyes absent homolog 3	down
Q9UQ13	Leucine-rich repeat protein SHOC-2	down
Q15643	Thyroid receptor-interacting protein 11	down
Q9BZI7	Regulator of nonsense transcripts 3B	down
Q6ZTQ3	Ras association domain-containing protein 6	down
H3BM91	COMM domain-containing protein 4 (Fragment)	down
E7EP72	UPF0449 protein C19orf25	down
Q9H832	Ubiquitin-conjugating enzyme E2 Z	down
Q9P086	Mediator of RNA polymerase II transcription subunit 11	down
Q2M296	Methenyltetrahydrofolate synthase domain-containing protein	down
B2RBA0	cDNA, FLJ95388, highly similar to Homo sapiens step II splicing factor SLU7 (SLU7), mRNA	down
O95498	Vascular non-inflammatory molecule 2	down
Q8TAV0	Protein FAM76A	down

B4DY64	cDNA FLJ52132, highly similar to Catechol-O-methyltransferasedomain-containing protein 1	down
A8K9A5	cDNA FLJ78114, highly similar to Homo sapiens thymidylate synthetase, mRNA	down
P20933	N(4)-(beta-N-acetylglucosaminyl)-L-asparaginase	down
Q86U28	Iron-sulfur cluster assembly 2 homolog, mitochondrial	down
Q9H8V3	Protein ECT2	down
E9PD50	Protein SMG7	down
Q96EK4	THAP domain-containing protein 11	down
A0A024R8N6	Thymidine kinase	down
O94919	Endonuclease domain-containing 1 protein	down
Q643R0	HCTP4	down
E7EW49	CLIP-associating protein 2	down
A8K099	cDNA FLJ77959, highly similar to Homo sapiens protein kinase, cAMP-dependent, regulatory, type I, beta (PRKAR1B), mRNA	down
Q71RC2	La-related protein 4	down
A0A024R7L2	HSPC142 protein, isoform CRA_a	down
Q96CX6	Leucine-rich repeat-containing protein 58	down
B3KP18	cDNA FLJ30946 fis, clone FEBRA2007622, highly similar to RAD50-interacting protein 1	down
M0R0P8	Unconventional myosin-IXb	down
A0A024QZY7	Geminin, DNA replication inhibitor, isoform CRA_a	down
